# Gut Microbiota-Related Effects of Tanhuo Decoction in Acute Ischemic Stroke

**DOI:** 10.1155/2021/5596924

**Published:** 2021-05-27

**Authors:** Qian Guo, Xiaoqing Jiang, Can Ni, Linjing Li, Li Chen, Yaqi Wang, Mo Li, Chunhui Wang, Li Gao, Huaiqiu Zhu, Juexian Song

**Affiliations:** ^1^State Key Laboratory for Turbulence and Complex Systems and Department of Biomedical Engineering, College of Engineering and Center for Quantitative Biology, Peking University, Beijing 100871, China; ^2^Department of Biomedical Engineering, Georgia Institute of Technology and Emory University, Georgia 30332, USA; ^3^Beijing Chaoyang Integrative Emergency Medical Center, Beijing 100000, China; ^4^Peking University-Tsinghua University-National Institute of Biological Sciences (PTN) Joint PhD Program, School of Life Sciences, Peking University, Beijing 100871, China; ^5^Department of Neurology, Xuanwu Hospital, Capital Medical University, Beijing 100053, China; ^6^Institute of Medical Technology, Peking University Health Science Center, Beijing 100191, China

## Abstract

Acute ischemic stroke (AIS) is a major cause of acquired adult disability and death. Our previous studies proved the efficacy and effectiveness of Tanhuo decoction (THD) on AIS. However, the therapeutic mechanism remains unclear. We recruited 49 AIS patients and 30 healthy people to explore the effects of THD+basic treatment on the poststroke gut microbiota of AIS patients using 16S rRNA sequencing, in which 23 patients received basic treatment (control group) and 26 patients received THD+basic treatment (THD group). By comparing the data before and after treatments, we found the THD group acquired better outcome than the control group on both clinical outcome indices and the characteristics of gut microbiota. In addition to the mediation on short-chain fatty acid- (SCFA-) producing bacteria in two groups, treatment in the THD group significantly decreased the lipopolysaccharide- (LPS-) producing bacteria to reduce LPS biosynthesis. Besides, the complexity of the cooccurrence of gut microbiota and the competition among LPS-producing bacteria and opportunistic pathogenetic bacteria were enhanced in the THD group. Treatment in the THD group also exhibited the potential in decreasing genes on the biosynthesis of trimethylamine (TMA), the precursor of Trimethylamine N-oxide (TMAO), and increasing genes on the degradation of TMA, especially increasing trimethylamine-corrinoid protein Co-methyltransferase (*mttB*) which catabolizes TMA to methane. These results hinted that THD+basic treatment might exert its efficacy by mediating the gut microbiota and microbial metabolites, including LPS and TMAO that aggravate the sterile inflammation and platelet aggregation. Moreover, the well-fitting regression model results in predicting the clinical outcome with the alteration of gut microbiota proved gut microbiota as a potential indicator of AIS and provided evidence of the communication between the gut and brain of AIS patients.

## 1. Introduction

Acute ischemic stroke (AIS) is a dominant cause of acquired adult disability and a major cause of death [1]. Nearly half of survivors lose self-care abilities and live with long-term health care [2]. The cost of poststroke health care brings a huge burden on economy, especially in the USA, where the expense per patient year exceeds the GDP per capita [3]. Large artery atherosclerosis and thrombosis are important causes of AIS, and the aggregation of platelet is a crucial step in thrombosis. Thrombolysis within six hours after symptom onset are proved efficient in clinic; however, it is limited by the narrow therapeutic time window and other constraints such as the history of intracranial surgery and the abnormalities of coagulation [4]. The disappointingly low thrombolysis rates have been reported in many countries, especially in the USA (2.4% in 2006) and China (1.6% in 2011) [5, 6]. In addition to the aggregation of platelet, sterile inflammation is another key step for the onset and aggravation of AIS. Acute sterile inflammation can disrupt blood-brain barrier and cause neuronal apoptosis after stroke and typically associates with a poor prognosis of AIS [7, 8]. In the treatment of AIS, except for arteriovenous thrombolysis, antiplatelet aggregation (Aspirin) (Class I; Level of Evidence A) and intravascular mechanical thrombectomy therapy, and other drugs, without sufficient evidence, have not been widely adopted by international stroke guidelines [9].

Both aggregations of platelet and sterile inflammation are closely associated with gut microbiota and microbial metabolites. Aberrant spreading of lipopolysaccharide (LPS), an important microbial metabolite, from gut will trigger a systemic immune response with an elevated level of proinflammatory cytokines, such as Tumor Necrosis Factor *α* (TNF-*α*), nuclear factor-*κ*B (NF-*κ*B), and Interleukin-6 (IL-6). Subsequently, the systematic release of proinflammatory cytokines will act on microglia to induce neuroinflammation, which will aggravate atherosclerosis and lead to a poor outcome of stroke [10, 11]. Besides, LPS interacts with platelet directly and then stimulates the secretion and aggregation of platelet synergizing with low concentrations of platelet agonists and therefore aggravates thrombus formation significantly [12]. Trimethylamine N-oxide (TMAO) is another critical gut microbiota-derived metabolite that has been proved to promote ischemic vascular diseases [13]. Gut microbiota generates trimethylamine (TMA), the precursor of TMAO, using phosphatidylcholine, lecithin, and L-carnitine from meat, seafood, and eggs, and TMA is later processed to TMAO in the liver. TMAO induces vascular inflammation via NF-*κ*B signaling and mitogen-activated protein kinase [14]. Additionally, TMAO can activate the stimulus-dependent platelet, and the aggregation of activated platelet and the generation of intra-arterial thrombus occur subsequently [15]. Both mechanistic experiments and epidemiological studies support that TMAO is a biomarker of arteriosclerosis and the high plasma concentration of TMAO is an indicator of poor outcome of stroke [16–18]. The inflammation and aggregation of platelet caused by either LPS or TMAO promote each other [19, 20], which is potential to accelerate thrombosis and ischemic injury. As gut microbiota participates in the bidirectional communication between the gut and the brain and modulates the hemostasis of hosts, the microbiota-gut-brain axis is fulfilled and plays an important role in brain injury, inflammation, and brain diseases [11, 21]. With the substantial enhancement of perception on the roles of gut microbiota and microbial metabolites in central nervous diseases, gut microbiota has been proposed as a therapeutic target in these diseases, including stroke, which has led to the analysis of gut microbiome in more and more studies [22].

Due to the unsatisfactory effectiveness of basic treatment with Western medicine (WM) on AIS [23], including drugs for antiplatelet (Aspirin, Clopidogrel), lowering lipid (Statins), and improving circulation (Edaravone, Alprostadil, Ureclin, and Butylphthalide), people engage in developing new drugs targeting on immune response to perform neuroprotective treatment on AIS patients [24] and also turn to the traditional Chinese medicine (TCM). TCM can remodel gut microbiota and downregulate inflammation [25, 26] and has been proved to be an efficient intervention on stroke since 1999 or even earlier [27, 28]. Our previous studies proved the satisfactory outcome of Tanhuo decoction (THD) on AIS, and no adverse side effects were found in clinic [23]. And by quantitative analysis, we found that THD could decrease the levels of several inflammatory factors, including Fib (fibrinogen), PAgT (platelet aggregation test), CRP (C-reactive protein), and TMAO, in the plasma of AIS patients. Even though that study is an important step forward to reveal the role of TCM on AIS, there is still a large gap between the therapeutic mechanisms and the effects of TCM on AIS. Meanwhile, the related studies on TCM, including THD, have paid little attention to gut microbiota.

In this study, we included 49 AIS patients and 30 healthy people as the research objects. The AIS patients were randomly divided into the control group (basic treatment using WM, *n* = 23) and the THD group (THD+basic treatment, *n* = 26), respectively. The 16S rRNA sequence data of gut microbiota and clinical information before and after the treatments were utilized to explore the effects of the two treatments on gut microbiota of AIS patients. Comparing clinical outcome indices of AIS patients in the two groups, we found that the THD group got better outcomes compared to the control group. With the comparative analysis of 16S rRNA sequences of gut microbiome before and after treatments, we found that gut microbiota of the THD group became closer to that of health samples than those of the control group. In addition to the regulation on short-chain fatty acid- (SCFA-) producing bacteria by both two treatments, the basic treatment decreased the relative abundance of secondary bile acid-producing bacteria, *Dorea*. By contrast, THD+basic treatment significantly reduced the LPS-producing bacteria to decrease the level of LPS biosynthesis. According to the quantitative analysis of the predicted genes, it also had the potential to decrease genes on the biosynthesis of TMA and increase genes on the degradation of TMA and especially increase trimethylamine-corrinoid protein Co-methyltransferase (*mttB*) that catabolizes TMA to methane. Furthermore, the treatment in the THD group enhanced the complexity of the cooccurrence network of gut microbiota, reconstructed the correlations among SCFA-producing genera, and promoted the competition among opportunistic pathogenetic bacteria and LPS-producing bacteria. These results reveal the gut microbiota-related effects of THD on AIS and provide a basis to disclose the therapeutic mechanism of THD on AIS from the perspective of microbiota-gut-brain axis.

## 2. Materials and Methods

### 2.1. Ethical Approval

The study was approved by the Ethics Committee of Xuanwu Hospital Capital Medical University, and all participants signed informed consent.

### 2.2. Inclusion and Exclusion Criteria

From June 16, 2018, to June 8, 2019, 49 AIS patients and 30 healthy participants were recruited. All AIS patients were included with the following criteria: (a) met the diagnostic criterion for ischemic cerebrovascular disease at the Fourth National Cerebrovascular Disease Conference; (b) diagnosed with head CT or MRI examination within 3 days after the onset of stroke; (c) associated with large artery atherosclerosis defined by Trial of Org 10172 in Acute Stroke Treatment (TOAST); (d) examined by ultrasound on carotid and transcranial color-coded duplex (TCCD) ultrasound; (e) stable vital signs and normal heart, liver, and kidney functions; (f) enrolled within 7 days from the onset of symptom; and (g) with complete clinical information. The AIS patients were also screened with the following exclusion criteria: (a) diagnosed as intracranial hemorrhage or nonischemic disease based on CT or MRI; (b) indications for anticoagulation therapy (cardiogenic embolism, such as atrial fibrillation, and myocarditis); (c) receiving intravenous thrombolytic therapy or interventional therapy after the onset this time; (d) with contraindications to Aspirin; (e) used antibiotics or other drugs known to affect gut microbiota for coinfection after enrollment; (f) arrangement for recanalization (interventional surgery or vascular surgery) within 3 months; (g) pregnant, lactating women or women of childbearing age who have a pregnancy plan within 3 months; (h) severe heart, liver, and kidney dysfunction; (i) with imperfect clinical data and specimen collection; and (j) participated in clinical research of other drugs and equipment simultaneously. The healthy participants included in this study met the following criteria: (a) free of any organic diseases or underlying diseases; (b) examined by carotid ultrasound; (c) match AIS patients with regard to basic characteristics, such as gender, age, and height weight; and (d) no antibiotics or probiotics intake during the sampling period.

### 2.3. Therapeutic Methods

In this prospective observational study, 49 AIS patients admitted in our hospital were enrolled from June 2018 to June 2019. 30 people meeting the inclusion criteria of healthy participants were included in the health group. All patients were separated into two groups randomly. One group was treated with basic treatment (control group, *n* = 23), and another was given THD+basic treatment (THD group, *n* = 26). Basic treatment contains drugs for antiplatelet (Aspirin and Clopidogrel), lowering lipid (Statins), and improving circulation (Edaravone, Alprostadil, Ureclin, and Butylphthalide). Each unit of THD contains 9 g Coptidis Rhizoma, 5 g Rhei Radix and Rhizoma, 9 g Lophatherum Gracile, 9 g Forsythia, and 9 g Bile Arisaema. All these herbs used in the current study were purchased from Sinopharm Group Beijing Huamiao Pharmaceutical Co., Ltd. The main chemicals of herbs were determined by Sinopharm Group Beijing Huamiao Pharmaceutical Co., Ltd., via high performance liquid chromatography. The batch number and the content of the main chemicals of each herb are listed in Supplementary Table 1. All the herbs were conformed to meet the standards of China Pharmacopoeia (2015 edition).

THD was given as decoction in THD group, and it was prepared by Xuanwu Hospital Capital Medical University according to a standard production process as follows: (a) put each unit of THD herbs into a casserole; (b) add 600 ml of cold water (1 : 15, *w*/*v*), and soak for 20-30 minutes; (c) boil the soaked herbs for 2 hours, and filtrate the decoction using gauze; (d) add 10 times of the residue material weight of warm water, boil the mixture, and filter the gruff three times using gauze, for another 2 hours; and (e) mix the twice filtered decoction. Each dose of THD yielded 100 ml decoction. AIS patients in the THD group taken 1 dose decoction a day, half for morning and half for evening, for 7 days orally. We collected the clinical characteristics of the AIS patients before and after the treatments and those of the healthy participants.

### 2.4. Outcome Measures

The National Institute of Health stroke scale (NIHSS) scores, modified Rankin Scale (mRS) scores, Barthel Index (BI) scores, and the fire-heat scores were recorded before and after the two treatments. The neurologic impairment of AIS patients was quantified with NIHSS, a tool scoring the impairment caused by stroke from 0 to 42, where the scores ≤ 4 represent no symptoms or mild deficit and the scores > 4 represent moderate or severe deficit. The disability and ability of AIS patients were measured by mRS and BI, respectively. mRS runs from 0 to 6, representing health condition with no symptoms to death. BI ranges from 0 to 100, representing total dependence to independence in personal activities. Additionally, fire-heat scores were used to evaluate the syndromes of fire-heat including constipation, dry mouth, yellow urine, and red tongue in TCM [23]. The clinical outcome of stroke was represented by the reduction of NIHSS, mRS, and fire-heat score and the increment of BI (NIHSS-, mRS-, fire-heat score-, BI+).

### 2.5. Stool Sample Collection, DNA Extraction, and 16S rRNA Sequencing

Stool samples were collected from 30 controls and 49 AIS patients before and after treatments. Microbial community genomic DNA from the fresh stool samples was extracted using the E.Z.N.A.® soil DNA Kit (Omega Bio-tek, Norcross, GA, U.S.) in accordance with the manufacturer's instructions. The concentration and purity of DNA were monitored on 1% agarose gels. The primer pairs 338F (5′-ACTCCTACGGGAGGCAGCAG-3′) and 806R (5′-GGACTACHVGGGTWTCTAAT-3′) were selected to amplify the V3-V4 regions of the 16S rRNA gene in the extracted DNA. The PCR amplification procedure of 16S rRNA was set as follows: 95°C for 3 min (initial denaturation); 27 cycles of 95°C for 30 s (of denaturing), 55°C for 30 s (annealing), and 72°C for 45 s (extension); and 72°C for 10 min (extension), and stored at 4°C. Then, the PCR production was paired-end sequenced on Illumina MiSeq PE300 platform (Illumina, San Diego, USA).

### 2.6. 16S rRNA Amplicon Data Processing

The demultiplexed paired-end sequences were imported into Qiime2 version 2020.8.0 (Qiime2) [29]. Quality control and the construction of the feature table of the amplicon sequence variant (ASV) were carried out with a DADA2 pipeline with the following parameter setting: –p-trunc-len-f 295, –p-trunc-len-r 295, and default values for other parameters. ASVs were taxonomically annotated using a naive Bayes classifier pretrained with 99% identity Greengenes rRNA database (Version 13.8.99) [30]. After that, the abundance matrices at the levels of phylum, class, order, family, and genus were created for each sample [31]. The abundance of bacteria before and after treatments was compared using the Wilcoxon rank sum test. We used the gut microbiota of the health group to represent the healthy status of gut microbiota and characterized the health degree of gut microbiota of AIS patient with the Euclidean distance between the relative abundance matrix of gut microbiota of AIS patient and that of the centroid of health group. The higher Euclidean distance meant the lower health degree. In order to describe the improvement of gut microbiota status using the two treatments, we calculated the increment of such Euclidean distances (IED) after the treatment. The negative IED represented the positive improvement on gut microbiota. Alpha diversity (Shannon diversity and Chao1) within a sample was calculated using *phyloseq* R package (version 1.32.0) [32]. While Chao1 index reflects the richness of gut microbiota, Shannon index measures both the richness and evenness. The cooccurrences among bacteria were calculated with Python-based SparCC [33] tool using the SparCC correlation method. Cooccurrence with an absolute SparCC correlation coefficient larger than 0.4 and the corresponding *Q* ≤ 0.05 was considered significant. The cooccurrence networks were visualized using Cytoscape (version 3.8.0) [34]. The hub bacteria were identified using Cytohubba plugin [35] based on Maximal Clique Centrality (MCC) algorithms. The positive connections, negative connection, complexity, and degrees of nodes were used to describe the topological characteristics of the cooccurrence network [36, 37]. To explore the functional capacity of participants' gut microbiota, we applied the PICRUST2 pipeline [38] to predict the KEGG Orthology (KO) profile for each sample from the relative abundances of ASVs. Bacterial contributions on specific KO were also output by PICRUST2. KEGG pathway profile was aggregated from KO profile. The Linear Discriminant Analysis Effect Size programme (LEfSe; http://huttenhower.sph.harvard.edu/galaxy/) [39] was used to identify KEGG pathways that are significantly different before and after treatments. To acquire the KOs directly related to the metabolism of TMA without omission, R package KEGGREST (Tenenbaum D. KEGGREST: Client-side REST access to KEGG. R package version 1.30.1) was used. All the KEGG REACTIONs with TMA as reactant or product were traced, and then the KOs utilized in each tracked reaction were collected. The abundance of each KO was compared before and after treatments and test using the paired Wilcoxon rank sum test. Multivariate linear regressions with feature selection, using the lasso penalized maximum likelihood technique, were performed with the “glmnet” R package (version 4.0.2) [40]. All the figures were plotted using R (version 4.0.2).

### 2.7. Statistical Analysis

Continuous variables were represented by the mean ± standard deviation (SD). The two-tailed Student's *t*-test with Welch's correction was used for comparing normally distributed continuous variables, whereas the Wilcoxon rank sum test was used for nonnormally distributed continuous variables. Discrete variables were compared by the chi-square test or two-tailed Fisher's exact test. The comparison for a variable before and after treatments was a paired test. The *p* value outputs by statistical tests in this study were adjusted with false discovery rate (*Q*). *Q* ≤ 0.05 represented statistically significant.

## 3. Results

### 3.1. Better Outcome of THD Treatment Compared to Basic Treatment

49 recruited AIS patients underwent randomization (23 with basic treatment enrolled in the control group; 26 with THD+basic treatment enrolled in the THD group). Besides, 30 people meeting the inclusion criteria of health sample were included in the health group. The two treatment groups were generally matched on the baseline characteristics (*Q* > 0.05, Supplementary Table 2).

**Figure 1:**
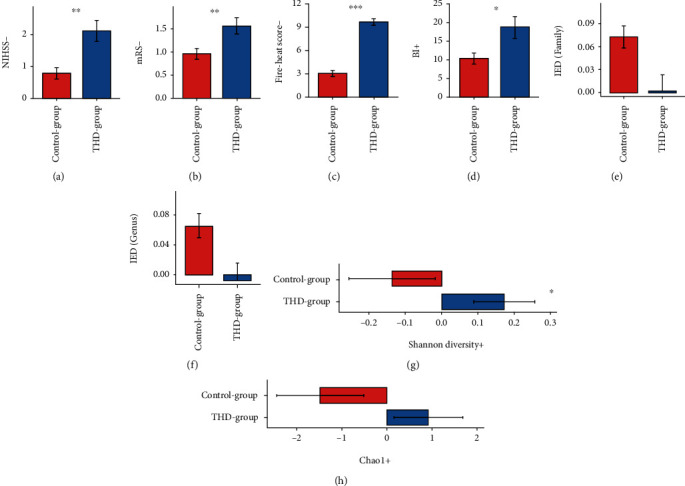
Comparison of outcome in control and THD groups. (a) Reduction of NIHSS in the two treatment groups. NIHSS was reduced more in the THD group than in the control group (Wilcoxon rank sum test, *Q* = 0.0012, 0.783 ± 0.85 (control) vs. 2.111 ± 1.761 (THD)). (b) Reduction of mRS in the two treatment groups. mRS was reduced more in the THD group than in the control group (Wilcoxon rank sum test, *Q* = 0.01, 0.957 ± 0.562 (control) vs. 1.556 ± 0.892 (THD)). (c) Reduction of fire-heat score in the two treatment groups. Fire-heat score was reduced more in the THD group than in the control group (Wilcoxon rank sum test, *Q* = 0, 3.13 ± 1.89 (control) vs. 9.704 ± 2.35 (THD)). (d) Increment of BI in the two treatment groups. BI was improved more in the THD group than in the control group (Wilcoxon rank sum test, *Q* = 0.022, 10.652 ± 7.12 (control) vs. 19.074 ± 15.382 (THD)). (e) IED to the health group for AIS samples after treatment (family level). Gut microbiota of AIS patients in the THD group became closer to health samples than those in the control group on family level (Wilcoxon rank sum test, *Q* = 0.053, 0.073 ± 0.137 (control) vs. 0.002 ± 0.109 (THD)). (f) IED to the health group for AIS samples after treatment (genus level). Gut microbiota of AIS patients in the THD group became closer to health samples than those in the control group on genus level (Wilcoxon rank sum test, *Q* = 0.077, 0.064 ± 0.154 (control) vs. −0.008 ± 0.118 (THD)). (g) Increment of alpha diversity (Shannon) of AIS samples. In THD group, the Shannon indices of AIS samples were increased, while they were decreased in the control group (Wilcoxon rank sum test, *Q* = 0.04, −0.136 ± 0.571 (control) vs. 0.172 ± 0.428 (THD)). (h) Increment of alpha diversity (Chao1) of AIS samples. In THD group, the Chao1 indices of AIS samples were increased, while they were decreased in the control group (Wilcoxon rank sum test, *Q* = 0.056, −1.478 ± 4.611 (control) vs. 0.923 ± 3.888 (THD)). Shannon diversity +: the increment of Shannon diversity; Chao1 +: the increment of Chao1. ^∗^*Q* ≤ 0.05, ^∗∗^*Q* ≤ 0.01, and ^∗∗∗^*Q* ≤ 0.001.

With the unbiased duration of treatment, patients in the THD group achieved better outcomes than basic treatment with regard to both the clinical outcome of stroke and the improvement of gut microbiota (Figure 1). The clinical outcome of stroke was represented by the reduction of NIHSS, mRS, and fire-heat score and the increment of BI (NIHSS-, mRS-, fire-heat score-, and BI+). In the THD group, NIHSS-, mRS-, fire-heat score-, and BI+ were 2.111 ± 1.761, 1.556 ± 0.892, 9.704 ± 2.35, and 19.074 ± 15.382, which were all significantly larger than those in the control group (0.783 ± 0.85, 0.957 ± 0.562, 3.13 ± 1.89, and 10.652 ± 7.12, Figures 1(a)–1(d)). Besides, in order to describe the change of health degree of gut microbiota of AIS patients after the two treatments, we calculated IED (Materials and Methods) of each AIS patient. While IEDs in the control group were positive, IEDs in the THD group were negative and significantly lower. The results of IED were consistent at genus (Figure 1(e)) and family (Figure 1(f)) levels. We thus inferred that THD+basic treatment better improved the health degree of gut microbiota community. In addition to IED, we investigated the changes on dominant bacteria (top 5 abundant families/genera in each sample group), which contributed more to IED than low abundance bacteria. We calculated the absolute differences in the abundance of dominant bacteria between AIS patients and health samples and compared the changes of the absolute differences after treatment between the two treatment groups (Supplementary Figure 1). We found that treatment in the THD group could shrink such differences on the most abundant bacteria, including *Prevotellaceae* (family), *Bacteroidaceae* (family), *Prevotella* (genus), and *Bacteroides* (genus), between AIS patients and health samples; however, the treatment in the control group played the opposite role. It is noteworthy that *Bacteroidaceae*, including *Bacteroides*, are important LPS-producing bacteria. Furthermore, while the alpha diversity (Shannon diversity and Chao1) of gut microbiota was lowered in the control group, it was significantly enhanced in the THD group (Figures 1(g) and 1(h)), which meant THD+basic treatment could lead to a richer and more balanced gut microbiota community compared to basic treatment.

### 3.2. Shifts of Gut Microbiota in the Control Group

**Figure 2:**
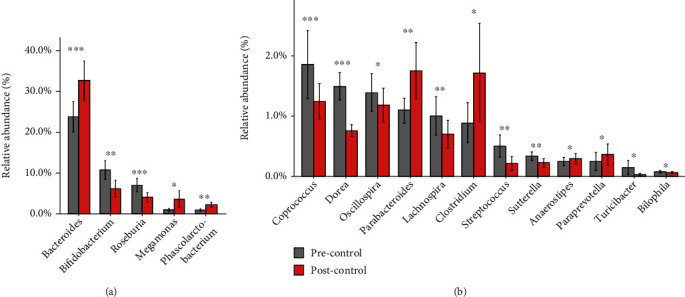
Relative abundance of differential genera pre- and posttreatment in the control group. 17 genera varied significantly after treatment in the control group, where seven genera decreased and 10 genera increased. (a) Differential genera, with relative abundance more than 2% either pretreatment or posttreatment. (b) Differential genera, with relative abundance less than 2% both pretreatment and after treatment. ^∗^*Q* ≤ 0.05, ^∗∗^*Q* ≤ 0.01, and ^∗∗∗^*Q* ≤ 0.001. Precontrol: pretreatment samples in the control group; Postcontrol: posttreatment samples in the control group.

Significant variations of bacterial abundance occurred with basic treatment. After basic treatment, the number of genus decreased, with 14 genera disappearing and eight genera appearing (Supplementary Figure 2A). The abundance of 17 genera changed significantly with basic treatment (*Q* ≤ 0.05), where seven genera increased and 10 genera decreased (Figure 2). Two LPS-producing genera (*Bacteroides* and *Oscillospira*) significantly altered. While the predominant genus, *Bacteroides*, increased, *Oscillospira* with relatively lower abundance decreased. Additionally, nine of the SCFA-producing genera altered remarkably. Exactly, butyrate-producing genera changed inconsistently, with two genera (*Anaerostipes* and *Gemmiger*) increasing and three (*Coprococcus*, *Roseburia*, and *Lachnospira*) decreasing. A propionate-producing genus, *Phascolarctobacterium*, increased, whereas acetate-producing genera, including *Bifidobacterium* and *Streptococcus*, reduced concurrently. Furthermore, *Dorea*, related to secondary bile acid biosynthesis and glucose metabolism [41], decreased after basic treatment.

**Figure 3:**
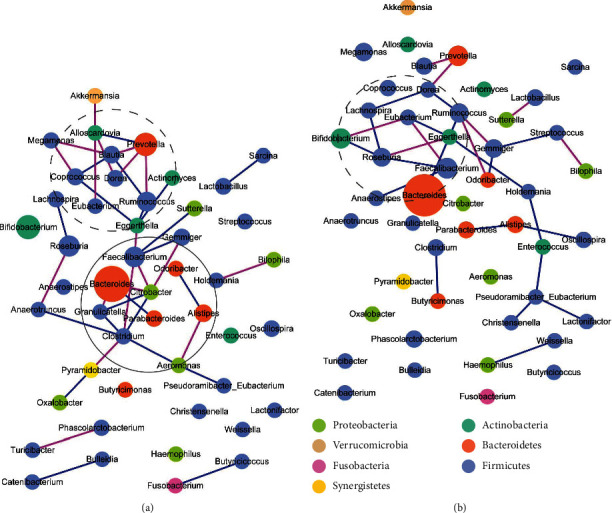
Bacterial co-occurrence network pre and post treatment in control group. (a) Co-occurrence network of genera before treatment in control group. The network was dominated by a chain of hub genera, including *Clostridium*, *Citrobacter*, *Faecalibacterium*, *Eggerthella*, *Ruminococcus*, *Blautia*, *Prevotella*, *Alloscardovia*, *Roseburia*, *Coprococcus*, *etc*. *Bacteroides*, an LPS-producing genus, competed with two genera and was complementary to one genus. (b) Co-occurrence network of genera after treatment in control group. In control group, the co-occurrence network was simplified after treatment, characterized by the reduced connections and complexity compared to the network before treatment. The chain appearing before treatment disappeared after treatment and a closed loop predominant by SCFA-producing genera occurred. *Bacteroides* became to collaborate with other genera. Red line: negative correlation; Blue line: positive correlation; the color of nodes stands for the phylum of the genus; the size of a node stands for the average abundance of the genus; the ellipses with different lines represent different clusters.

The variation of bacterial abundance in control group simplified the cooccurrences among bacteria (Figure 3). After basic treatment in the control group, the cooccurrence network, composed of significant correlations (Materials and Methods), was weakened, containing 19 positive and nine negative cooccurrences with reduced complexity compared to the pretreatment network (259.287 vs. 418.706) (Supplementary Table 3). The pretreatment network was dominated by a chain of hub genera (Supplementary Table 4,5), including *Clostridium*, *Citrobacter*, *Faecalibacterium*, *Eggerthella*, *Ruminococcus*, *Blautia*, *Prevotella*, *Alloscardovia*, *Roseburia*, and *Coprococcus*. In the posttreatment network, with the obvious reduction of connections, the chain was broken. The cooccurrence network after basic treatment was rebuilt and became dominant by a closed loop composed of *Ruminococcus*, *Dorea*, *Lachnospira*, *Roseburia*, *Eggerthella*, *Bifidobacterium*, *Faecalibacterium*, and *Eubacterium*, in which SCFA-producing genera took a large proportion (6/8). Besides, the cooccurrence loop associated with other SCFA-producing genera, including *Odoribacter*, *Blautia*, *Gemmiger*, and *Streptococcus*. The closed loop and its connected SCFA-producing bacteria possibly served as a functional group. Furthermore, *Bacteroides*, the LPS-producing bacteria significantly increasing after basic treatment, cooccurred with different bacteria in pretreatment and posttreatment networks. In the pretreatment network, *Bacteroides* negatively connected with two genera, *Citrobacter* and *Granulicatella*, and positively connected with *Parabacteroides*. In the posttreatment network, *Bacteroides* only positively correlated with another genus, *Ruminococcus*. As proposed in a previous study [42] that the positive and negative correlations represent metabolic complementarity and competition, respectively, it hinted that there was probably less competition for LPS-producing bacteria after basic treatment.

**Figure 4:**
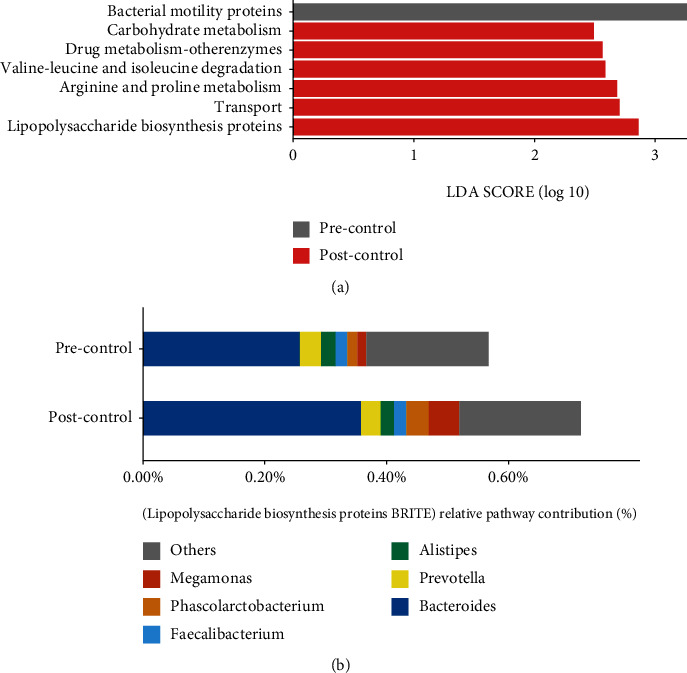
Significantly different KEGG pathways and comparison of bacterial contribution on “Lipopolysaccharide biosynthesis proteins” KEGG BRITE between pretreatment and posttreatment samples in the control group. (a) Significantly different KEGG pathways between pretreatment and posttreatment samples in the control group using Linear discriminant analysis effect size (LEfSe) analysis (>2 log (LDA score)). (b) Relative bacterial contribution on “Lipopolysaccharide biosynthesis proteins” KEGG BRITE before and after treatment in the control group. “Lipopolysaccharide biosynthesis proteins” KEGG BRITE enriched after treatment in the control group. Three significant increasing genera, including *Bacteroides*, *Phascolarctobacterium*, and *Meganomas*, contributed more to “Lipopolysaccharide biosynthesis proteins” pathway after treatment. The top five bacterial donators in each group are shown in detail, and others were aggregated into others. Precontrol: pretreatment samples in the control group; Postcontrol: posttreatment samples in the control group.

By analyzing the KEGG Orthology (KO) and KEGG pathway predicted from 16S rRNA sequences using PICRUST2 [38] (Materials and Methods), we found the enrichment of seven pathways or brites altered significantly after basic treatment in control group (Linear discriminant analysis (LDA) score > 2) (Figure 4(a)). Remarkably, “Lipopolysaccharide biosynthesis proteins” KEGG BRITE (BR: ko01005) enriched in posttreatment samples, which hinted that basic treatment might not inhibit the biosynthesis of bacteria-derived LPS. Analyzing the contribution of genera on “Lipopolysaccharide biosynthesis proteins” BRITE, we found *Bacteroides* contributed the most both pre- and posttreatments. The increased level of “Lipopolysaccharide biosynthesis proteins” BRITE in posttreatment samples was related to the enhanced contribution of three remarkably increased genera, including *Bacteroides*, *Phascolarctobacterium*, and *Meganomas* (Figure 4(b)).

In summary, the above results suggested that basic treatment decreased the secondary bile acid-producing bacteria, *Dorea*, and regulated several SCFA-producing bacteria. These are coincident with former researches on the medicine used in basic treatment. The antiplatelet-aggregation drug, Aspirin, was reported to reduce *Dorea* [43]. The cholesterol-lowering drugs, such as Atorvastatin and Rosuvastatin, were reported to increase SCFA-producing genera [44]. The decrease of secondary bile acid will downregulate the reabsorption of bile acid. And SCFA can lower immunomodulatory and anti-inflammatory in the gut [45, 46]. So, decreasing secondary bile acid-producing bacteria and regulating several SCFA-producing bacteria are probable processes for basic treatment to alleviate stroke. However, as the predominant LPS-producing bacteria, *Bacteroidetes*, and the level of “LPS biosynthesis proteins” BRITE were increased, and the competition for LPS-producing bacteria was reduced posttreatment; it hinted that the basic treatment was probably less efficient to inhibit LPS and the related sterile inflammation, which needed to be quantitatively analyzed in the future study.

### 3.3. Shifts of Gut Microbiota in the THD Group

**Figure 5:**
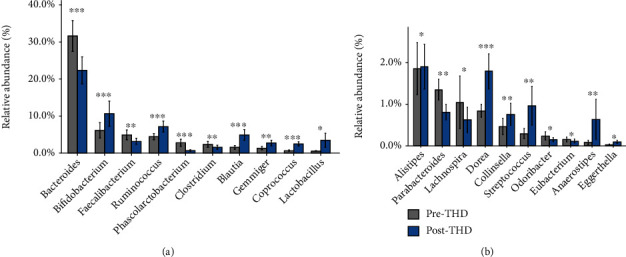
Relative abundance of differential genera pre and post treatment in the THD group. (a) Differential genera, with relative abundance more than 2% either pretreatment or posttreatment. (b) Differential genera, with relative abundance less than 2% both pretreatment and after treatment. ^∗^*Q* ≤ 0.05, ^∗∗^*Q* ≤ 0.01, and ^∗∗∗^*Q* ≤ 0.001. Pre-THD: pretreatment samples in THD group; Post-THD: posttreatment samples in THD group.

Significant shifts of bacteria also occurred after treatment in the THD group. The number of genus increased with 13 genera appearing and four disappearing (Supplementary Figure 2B). The abundance of 20 genera changed significantly (*Q* ≤ 0.05), where 11 genera increased and nine genera decreased (Figure 5). Remarkably, *Bacteroidetes*, the LPS-producing genus, decreased significantly. Another LPS-producing genus, *Oscillospira*, also decreased (*Q* = 0.072). Besides, 14 of the 20 differential genera were related to the biosynthesis of SCFA. Several butyrate-producing genera altered significantly. While *Anaerostipes*, *Gemmiger*, and *Coprococcus* increased, *Eubacterium*, *Lachnospira*, and *Odoribacter* decreased. *Phascolarctobacterium*, a propionate-producing genus, reduced significantly. Reversely, acetate-producing genera, including *Bifidobacterium*, *Blautia*, *Ruminococcus*, and *Streptococcus*, increased simultaneously.

**Figure 6:**
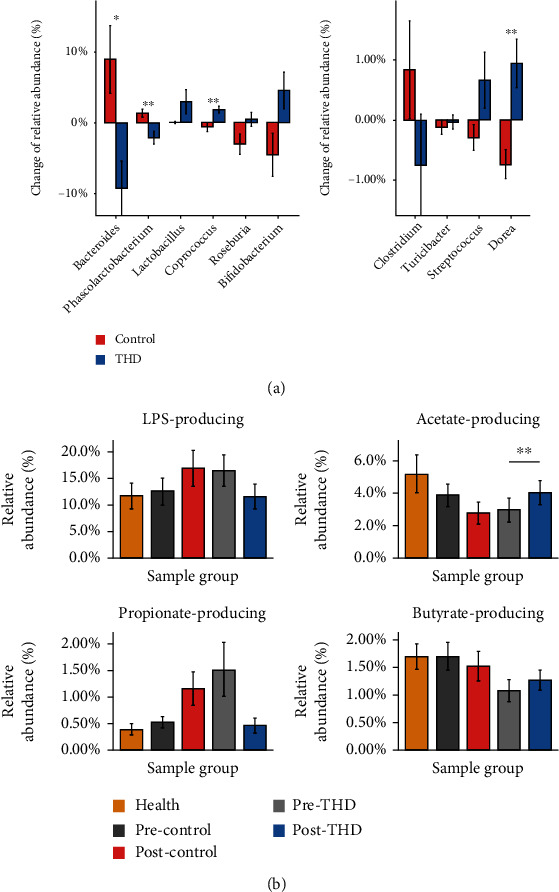
Change of genera shifting differently between two treatment groups and comparisons on LPS- and SCFA-producing bacteria among different sample groups. (A1, A2) Change of genera that shifted differently between two treatment groups (*p* value ≤ 0.05). (A1) Genera, with relative abundance more than 1% either pretreatment or after treatment and shifted differently between two treatment groups. (A2) Genera, with relative abundance more than 1% either pretreatment or after treatment and shifted differently between two treatment groups. (b) Comparisons on LPS- and SCFA-producing bacteria among AIS groups and health group. The abundance sums of LPS-producing bacteria, acetate-producing bacteria, and propionate-producing bacteria in the post-THD group were closer to those of health group. The abundance sum of butyrate-producing bacteria was slightly increased in the post-THD group, while it was decreased to some extent in the postcontrol group. ^∗^*Q* ≤ 0.05, ^∗∗^*Q* ≤ 0.01, and ^∗∗∗^*Q* ≤ 0.001. Precontrol: pretreatment samples in control group; Postcontrol: posttreatment samples in control group; Pre-THD: pretreatment samples in THD group; Post-THD, posttreatment samples in THD group. Precontrol and pre-THD were distinguished by dark grey and light grey in the figure.

Comparing the shifts of gut microbiota in the THD group and control group, we found four genera shifted divergently (*Q* ≤ 0.05) in the two treatment groups (Figure 6 A1 and A2). Noteworthy, *Bacteroides* (LPS-producing genus) was changed oppositely in the two groups. The result hinted at the possible better effect of THD+basic treatment on downregulating the biosynthesis of LPS compared to basic treatment. However, the inference needs to be further proved by the analysis of LPS related pathways and experimental data. The same happened on *Phascolarctobacterium* (propionate-producing bacteria), *Coprococcus* (butyrate-producing bacteria), and *Dorea*. Furthermore, we compared the gut microbiota of AIS patients to health samples. We focused on the LPS- and SCFA-producing bacteria, and aggregated them into LPS-producing, acetate-producing, propionate-producing, and butyrate-producing bacteria. Interestingly, compared to posttreatment samples in the control group, the abundance sums of LPS-producing bacteria, acetate-producing bacteria, and propionate-producing bacteria in the posttreatment samples in the THD group were closer to those of the health group (Figure 6(b)). Besides, after treatment, the abundance sum of butyrate-producing bacteria was increased in the THD group, while they were decreased to some extent in the control group. These comparison results additionally demonstrated the positive effects of THD on gut microbiota. The detailed comparison results of each LPS-/SCFA-producing bacterium are shown in Supplementary Figure 3.

**Figure 7:**
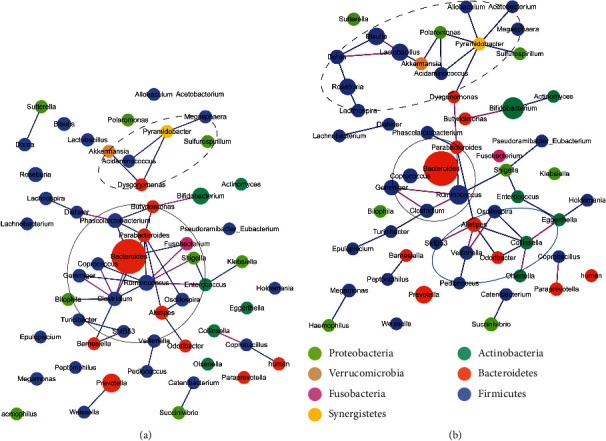
Bacterial cooccurrence network pre- and posttreatment in the THD group. (a) Cooccurrence network of genera before treatment in the THD group. The network contained a big cluster, dominant by *Clostridium*-*Ruminococcus*-*Parabacteroides* cooccurrence cluster, and a small cluster centered on *Pyramidobacter*. Most of the SCFA-producing bacteria were contained in the two clusters. *Bacteroides*, an LPS-producing bacterium, competed with one genus and was complementary with two genera. (b) Cooccurrence network of genera after treatment in THD group. The cooccurrence network became more complicated after treatment, with more connections and higher complexity. While the *Clostridium*-*Ruminococcus*-*Parabacteroides* dominant cluster shrank caused by the significant increment of *Ruminococcus*, the *Pyramidobacter* dominant cluster enlarged and linked more SCFA-producing bacteria, especially two acetate-producing genera, *Blautia* and *Acetobacterium*. *Bacteroides* lost all correlations with other genera after treatment. Red line: negative correlation; blue line: positive correlation; colors of nodes stand for the phylum of the bacteria; sizes of the bubbles stand for the average abundance; the ellipses with different lines represent different clusters.

These differential genera strengthened the cooccurrence network in the THD group (Figure 7). The network analyses demonstrated the considerable profit of THD with regard to the cooccurrences of genera. The posttreatment cooccurrence network contained 32 positive and 13 negative cooccurrences with an enhanced complexity of 382.379996, compared to the pretreatment network (313.282) (Supplementary Table 3). Remarkably, with the significant changes in the abundance of several genera, especially those identified as hub genera (Supplementary Tables 4,5) pre- and posttreatment in the THD group, including *Ruminococcus*, *Clostridium*, *Parabacteroides*, *Lactobacillus*, and *Blautia*, the cooccurrence network changed from a *Clostridium*-*Ruminococcus*-*Parabacteroides* cluster dominant network to a more dispersed one with a bias of dominant genera. In the *Clostridium*-*Ruminococcus*-*Parabacteroides* dominant network of pretreatment samples, the three genera united most of the SCFA-producing genera contained in the network, including *Coprococcus*, *Gemmiger*, *Butyricimonas*, *Bifidobacterium*, and *Oscillospira*. Besides, the LPS-producing genus, *Bacteroides*, and some pathogenetic genera, including *Shigella*, *Enterococcus*, and *Klebsiella* were included in the *Clostridium*-*Ruminococcus*-*Parabacteroides* cluster. Additionally, other SCFA-producing genera, including *Megasphaera* and *Akkermansia*, were joined by *Pyramidobacter*, another hub genus with a lower degree and betweenness. In the posttreatment network, the cluster held by *Clostridium*-*Ruminococcus*-*Parabacteroides* was weakened according to the significant increment of *Ruminococcus*, and the cooccurrences between *Ruminococcus* and three genera (*Clostridium*, *Gemmiger*, and *Shigella*) were reversed compared to the pretreatment network. All the cooccurrences of *Bacteroides* (an LPS-producing genus) disappeared. Moreover, *Pyramidobacter*, with the improved degree and MCC score, associated with a coalescence of SCFA-producing genera, including *Akkermansia*, *Lactobacillus*, *Blautia*, *Roseburia*, and *Acetobacterium*. Two acetate-producing genera, *Blautia* and *Acetobacterium* (a genus that appeared after THD treatment), were not contained in the pretreatment network; however, they were associated with other bacteria in the posttreatment network. Interestingly, the two genera were predicted to provide *mttB* gene that can catabolize TMA to methane. The *Pyramidobacter*-centered cluster was probably a functional cluster, in which the acetate-producing bacteria were connected by other SCFA-producing bacteria to lower the level of TMA. Furthermore, a new cluster appeared, where several pathogenetic genera and LPS-producing genera, including *Eggerthella*, *Collinsella*, *Alistipes*, *Veillonella*, *Olsenella*, and *Oscillospira*, competed with each other, and might play a role in downregulating the level of LPS.

**Figure 8:**
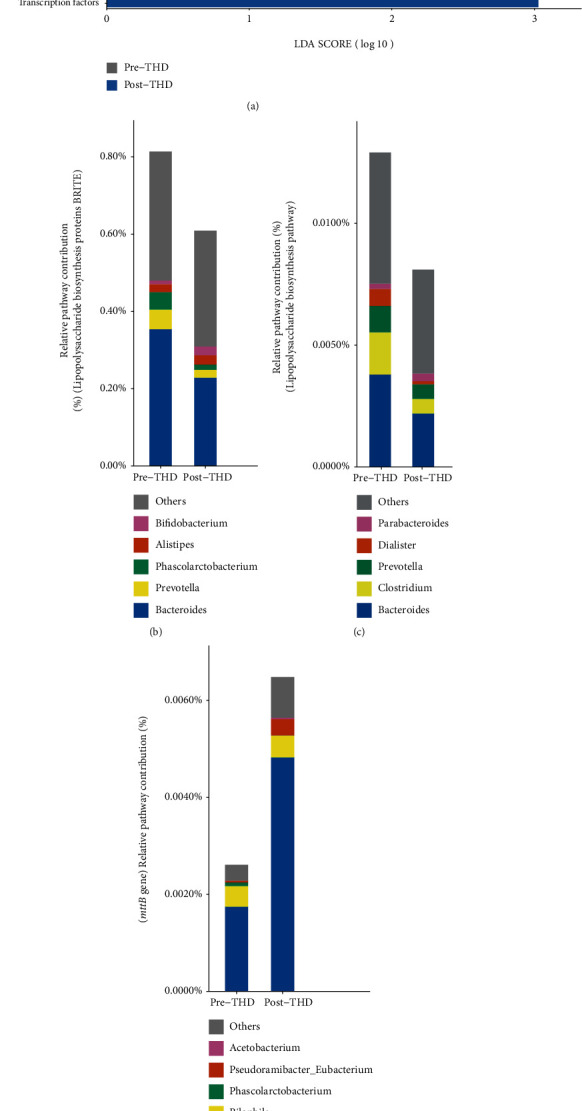
Significantly different KEGG pathways and comparisons of bacterial contribution on “Lipopolysaccharide biosynthesis proteins” KEGG BRITE, “Lipopolysaccharide biosynthesis” KEGG PATHWAY, and *mttB* gene between pretreatment and posttreatment samples in the THD group. (a) Significantly different KEGG pathways between pre- and posttreatment samples in the THD group using Linear discriminant analysis effect size (LEfSe) analysis (>2 log (LDA score)). (b) Relative bacterial contribution on “Lipopolysaccharide biosynthesis proteins” KEGG BRITE pre and post treatment in the THD group. “Lipopolysaccharide biosynthesis proteins” KEGG BRITE enriched before treatment in the THD group. *Bacteroides* and *Meganomas* contributed less on “Lipopolysaccharide biosynthesis proteins” KEGG BRITE after treatment. (c) Relative bacterial contribution on “Lipopolysaccharide biosynthesis” KEGG PATHWAY before and after treatment in the THD group. “Lipopolysaccharide biosynthesis” KEGG PATHWAY enriched before treatment. *Bacteroides*, *Clostridium*, *Prevotella*, and *Dialister* contributed less on “Lipopolysaccharide biosynthesis” KEGG PATHWAY after treatment. (d) Relative bacterial contribution on *mttB* gene before and after treatment in the THD group. *mttB* gene enriched after treatment. *Blautia* and *Acetobacterium* contributed more after treatment. The top five bacterial donators in each group are shown in detail, and others were aggregated into “Others.” Pre-THD: pretreatment samples in the THD group; Post-THD: posttreatment samples in the THD group.

Exploring the change of functional capacity of gut microbiota in the THD group, we conducted LEfSe analysis and identified 18 pathways altered significantly (LDA > 2, Figure 8(a)). Remarkably, “Lipopolysaccharide biosynthesis proteins” KEGG BRITE (BR: ko01005) and “Lipopolysaccharide biosynthesis” KEGG PATHWAY (PATH: ko00540) enriched in pretreatment samples (“Lipopolysaccharide biosynthesis proteins” KEGG BRITE: *Q* ≤ 0.05, LDA > 2; “Lipopolysaccharide biosynthesis” KEGG PATHWAY: *Q* ≤ 0.05), which hinted that the biosynthesis of bacteria-derived LPS was downregulated in the THD group. Analyzing the contribution of bacteria on “Lipopolysaccharide biosynthesis protein” BRITE and “Lipopolysaccharide biosynthesis” PATHWAY, we found *Bacteroides* contributed the most both before and after treatment. The lowered level of biosynthesis of LPS in posttreatment samples was related to the less contribution of *Bacteroides*, *Meganomas*, *Prevotella*, *Clostridium*, and *Dialister*, compared to pretreatment samples (Figures 8(b) and 8(c)). Additionally, we collected the TMA metabolic reactions and KOs. Eight KOs in six reactions were contained in our data. We compared the abundance of each KO before and after treatment and found that most (5/6) of KOs participating in the degradation of TMA were increased to some extent in the THD group, especially K14083 (*mttB*), which was upregulated significantly with *Q* ≤ 0.05 (Table 1). The KOs in the biosynthesis of TMA were decreased slightly (*Q* > 0.05) in the THD group. However, there was no significant change in all the related KOs in the control group. In our previous study, we found the plasma TMAO level of AIS patient was lowered significantly after THD+basic treatment with quantitative analysis [23], which is the bedrock and efficient evidence of our current study. So, it is reasonable to infer that regulating gut bacteria is a probable way for THD to lower the level of TMA and TMAO. To have a closer look at THD's regulation on gut bacteria that participated in the biosynthesis or degradation of TMA, a comparison of the bacterial contributions on TMA-related KOs before and after treatment was required. Here, we conducted such analysis on *mttB* gene, which was significantly upregulated in the THD group, as an example (Figure 8(d)). *Blautia* (acetate-producing genus) was the major genus donator both before and after treatment. The genus was considerably increased and donated more *mttB* genes after THD+basic treatment. *Acetobacterium*, another acetate-producing genus, uniquely appearing in the post-THD group, also contributed *mttB* gene. Remarkably, both *Blautia* and *Acetobacterium* were contained in the *Pyramidobacter* dominated cooccurrence cluster in the post-THD group and connected by other SCFA-producing bacteria (Figure 7).

Generally, the treatment in the THD group had several positive effects on the gut microbiota of AIS patients. It could significantly downregulate the biosynthesis pathway of LPS by inhibiting the LPS-producing bacteria. Besides, it led to the decrease of genes for the biosynthesis of TMA and the increase of genes for the degradation of TMA, especially the significant increase of *mttB* gene that degrades TMA to methane. Additionally, it remarkably increased acetate-producing bacteria, which were the major contributors to *mttB* gene. The treatment could also increase the cooccurrences among gut microbiota and promote the competition among LPS-producing bacteria and pathogenetic bacteria. In the THD group, in addition to basic treatment, patients were given THD, which mainly contains Coptidis Rhizoma, Rhei Radix and Rhizoma, Lophatherum Gracile, Forsythia, and Bile Arisaema, and the active ingredients include Berberine and Rhein. Berberine in Coptidis Rhizoma can lower the levels of blood glucose and LPS, inhibit several pathogenetic bacteria, and increase several SCFA-producing genera, including the acetate-producing genera *Ruminococcus* and *Blautia* [47–49]. Rhein in Rhei Radix and Rhizoma is reported to reduce serum uremic toxin and downregulate systemic inflammation and oxidative stress [50]. Rhein can also increase SCFA-producing bacteria and inhibit pathogenetic bacteria in gut [51]. In addition to these proofs which support our analysis results, our previous study, which found THD's inhibition of plasma TMAO level by quantitative analysis, is another efficient evidence for the gut microbiota-related effects of THD. So, it is reasonable to propose that THD can alleviate stroke via several probable approaches, including the inhibition of LPS-producing bacteria, the increase of acetate-producing bacteria, and the enhancement of cooccurrences among gut bacteria.

### 3.4. Alterations in the Gut Microbiota Can Be Used as a Prognostic Indicator of AIS

**Figure 9:**
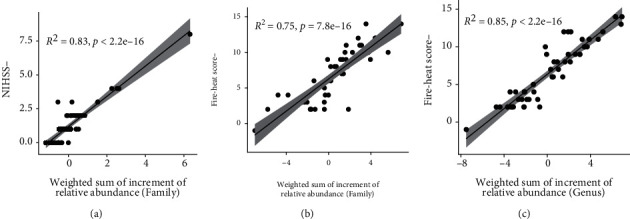
Scatter plots showing correlations of the increment of the abundance of bacteria with the clinical outcome of AIS. (a) Correlation between the increment of the abundance of bacteria (on family level) and the reduction of NIHSS. (b) Correlation between the increment of the abundance of bacteria (on family level) and the reduction of fire-heat scores. (c) Correlation between the increment of the abundance of bacteria (on genus level) and the reduction of fire-heat scores.

In this part, we explored the correlation between the clinical outcome of AIS and the changes in the abundance of gut microbiota. We performed multivariate linear regressions with feature selection, using the lasso penalized maximum likelihood technique in the “glmnet” R package (version 4.0.2). 22 bacterium families were selected to fit the change of NIHSS with the adjusted *R*-squared of 0.83, which meant the outcome was highly related to and could be efficiently reflected by the shifts of gut microbiomes (Figure 9(a)). Besides, we found the reduction of fire-heat scores was also perfectly fitted by the changes in the abundance of bacteria. The reduction of fire-heat score can be fitted by 23 families or 27 genera (Figures 9(b) and 9(c)) with the adjusted *R*-squared of 0.75 and 0.85, respectively. These consequences further demonstrated that gut microbiota is a potential indicator of the outcome of AIS. Remarkably, the coefficients of the LPS-producing genus *Bacteroides* and its family *Bacteroidaceae* were negative in all the linear analyses, which illustrated the aggravation of AIS by LPS-producing bacteria. These results additionally provided rationality for the hypothesis that it is a probable approach for THD+basic treatment to achieve a good outcome of AIS by inhibiting LPS-producing bacteria.

## 4. Discussion

AIS is a disease with high fatality rate and high disability rate. The cost of treatment and medical care of the disease brings a heavy economic burden to patients. As thrombolysis and Western Medicine are unsatisfactory [23] under several constraints, people turned to TCM and found that TCM led to a good outcome. However, the therapeutic mechanism of TCM, including THD, on AIS is unclear.

**Figure 10:**
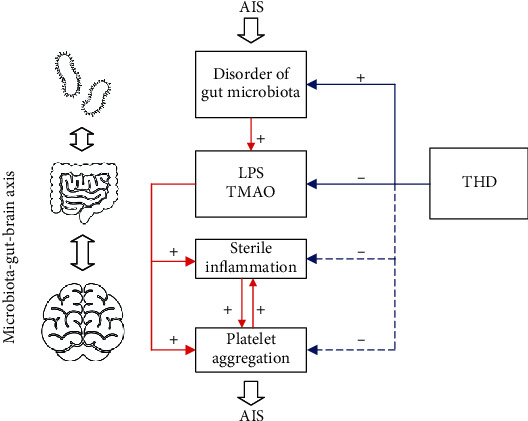
Hypothesis of the therapeutic mechanism of THD on AIS. The gut microbiota can be disrupted with the innervation of the dysfunctional nerve system in AIS patients. The disordered gut microbiota will produce LPS and the precursor of TMAO aberrantly. The sterile inflammation and platelet aggregation in the nerve system and vascular system, induced by LPS and TMAO, will aggravate AIS further. TCM will mediate the gut bacteria and subsequently reduce the levels of LPS and TMAO and then inhibit the aggravation of AIS. Blue solid line: included in our analysis results; blue dotted line: need to be verified further.

Sterile inflammation in the nerve system and vascular system and platelet aggregation are two pivotal steps in the onset and aggravation of AIS. Our previous study revealed that THD could effectively inhibit the inflammatory factors related to sterile inflammation and platelet aggregation in AIS patients, including TMAO, Fib, PAgT, and CRP [23]. This result was a prospective step forward in revealing the therapeutic mechanism of THD on AIS. The gut microbiota participates in the bidirectional communication between the brain and the gut in the brain-gut axis, and the disorder of microbial metabolites, especially LPS and TMA, can exacerbate sterile inflammation and platelet aggregation. In addition, studies on other diseases, such as type II diabetes [26], show that TCM mediates the metabolism and immunity of the host with the regulation of the gut microbiota and thereby achieves therapeutic effects. Furthermore, the effective ingredients, including Berberine and Rhein, in THD for stroke were reported to influence gut microbiota. Based on the pieces of evidence above, it is reasonable to assume that THD may treat AIS patients through the microbiota-gut-brain axis, where THD regulates several gut bacteria to lower microbial metabolites such as LPS and TMAO, which will lead to the downregulation of the sterile inflammation and platelet aggregation further (Figure 10). Driven by this hypothesis, we designed experiments, sampling strategies, and analysis procedures to explore the gut microbiome-related effects of THD on AIS and aimed to reveal the role of THD from the perspective of gut-brain communication in the microbiota-gut-brain axis.

As far as we know, this is the first study to investigate the effects of TCM on gut microbiota of AIS patients. In this study, we found that THD+basic treatment in the THD group fulfilled a better clinical outcome compared with basic treatment in the control group. Besides, in the THD group, the gut microbiota became closer to that of the health group than the control group. In addition to the mediation of SCFA-producing bacteria in both two groups, *Dorea*, the secondary bile-producing bacteria, was reduced in the control group, which may reduce the level of cholesterol subsequently. By contrast, the gut microbiota of AIS patients were altered with several specific characteristics in the THD group. After THD+basic treatment, there were a significant reduction of LPS-producing bacteria and the subsequent reduction in the biosynthesis of LPS. Besides, the cooccurrence network of gut microbiota was strengthened with more connections and higher complexity, and the competition among LPS-producing bacteria and pathogenetic bacteria was enhanced. Several clues to the inhibition of the level of TMA occurred, including the decrease of genes on the biosynthesis of TMA and the increment of genes on the decomposition of TMA, especially the remarkable increment on *mttB* gene which is utilized to catabolize TMA in the methanogenesis pathway. These clues coincided with the conclusion of our previous study that plasma TMAO level could be lowered by THD [23]. In the THD group, *mttB* gene was donated majorly by the acetate-producing bacteria that were increased significantly after treatment. It is noteworthy that the reported gut microbiota-related effects of active ingredients of THD can support our findings. Berberine was found to lower the levels of LPS and increase acetate-producing genera, including *Ruminococcus* and *Blautia* [47–49]. Rhein was also reported to increase several SCFA-producing bacteria and downregulate systemic inflammation [50, 51]. So, the analysis results in the current study provide a basis for the above assumption (Figure 10) and contribute to filling the gap between the therapeutic mechanism and effect of THD. Furthermore, by analyzing the correlations between the change of abundance of gut microbiota and the clinical outcome of AIS, we found the outcome of AIS (the reduction of NIHSS and the reduction of fire-heat score) could be well fitted by the change of abundance of bacteria on family or genus level. This result proves that the gut microbiota is an indicator of AIS and additionally provides rationality for the hypothesis that THD treatment achieves a good outcome of AIS by the regulation of the gut microbiota and the microbial metabolites involved in the microbiota-gut-brain axis.

In summary, our study revealed the gut microbiota-related effects of THD on AIS and provided a new clue to the therapeutic mechanism of THD on AIS. However, it is worth noting that there were slight differences between pretreatment samples in the control and THD groups caused by uncontrolled bias in random grouping and the relatively small sample size. The differences were embodied in both clinical outcomes and gut microbiota, especially cooccurrences among gut bacteria. On the one hand, the pretreatment samples of the THD group were evaluated with slightly higher NIHSS, mRS, fire-heat score, and lower BI (*Q* > 0.05, Supplementary Figure 4), showing that those samples might suffer more severe AIS. On the other hand, as shown in Supplementary Table 3, the complexity of the pretreatment cooccurrence network in the control group (418.706), which was closer to the health group (650.226), was higher than that of the pretreatment network in the THD group (313.282). However, the relatively more severe AIS and more disordered gut microbiota for samples before treatment did not weaken our conclusions about the better outcome in the THD group but strengthened it instead and hinted that THD+basic treatment may outperform basic treatment in treating patients with worse condition. Nevertheless, more unbiased data with a larger sample size is desired to further explore the effect of THD on gut microbiome and quantitatively evaluate the effectiveness of the gut microbiota as an indicator of AIS. Also, as 16S rRNA mainly provides taxonomy information of gut microbiota, more studies using whole metagenomic sequences, multiomics data, and the verification of biological experimental, are required.

## 5. Conclusions

This study revealed better outcomes in the THD group than in the control group and the alteration in gut microbiota of AIS patients in the THD group. Our results hinted that THD+basic treatment might exert its efficacy via the significant reduction of LPS-producing bacteria, the remarkable increment of acetate-producing bacteria, the enhanced complexity of cooccurrences of gut microbiota, and the strengthened competition of LPS-producing bacteria and opportunistic pathogens. These regulations are potential to inhibit the gut microbiota-derived metabolites, including LPS and TMAO which can aggravate the sterile inflammation and platelet aggregation. Moreover, this study also proved gut microbiota as a potential indicator of AIS and provided evidence of the communication between the gut and brain of AIS patients. These findings will help disclose the therapeutic mechanism of THD on AIS from the perspective of microbiota-gut-brain axis.

## Figures and Tables

**Table 1:** Changes of TMA-related KOs in two treatment groups.

Reaction	As reactant/product	Orthology	Control group	THD group
R02511	Reactant	K00317	↓	↑

R05623	Reactant	K18277	↑	↑

R09124	Reactant	K14082	↑	↓
		K14083	↓	↑
		K14084	↑	↑

R10016	Reactant	K14083	↓	↑∗

R07228	Reactant	K10670	↓	↑
		K21576	-	-
		K21577	-	-
		K21578	-	-
		K21579	-	-

R04877	Reactant	-	-	-

R10017	Reactant	-	-	-

R02560	Product	K07811	↓	↓
		K07812	↓	↓

R10285	Product	K20038	-	-

R11875	Product	K22443	-	-
		K22444	-	-

R11911	Product	K22443	-	-
		K22444	-	-

-: not appearing in our data; ↑: increased after treatment; ↓: decreased after treatment; ∗ means increased/decreased significantly with *Q* ≤ 0.05.

## Data Availability

All data were deposited to the NCBI SAR under BioProject number PRJNA683157.

## Supplementary Materials

Supplementary Materials Supplementary Table 1: batch numbers and components of the herbs in THD. Supplementary Table 2: baseline characteristics pretreatment in the control and THD groups. Supplementary Table 3: parameters of cooccurrence networks of all groups in this study. Supplementary Table 4: degrees of the genera in cooccurrence networks of patient groups in this study. Supplementary Table 5: MCC scores for Hub genera in cooccurrence networks of patient groups in this study. Supplementary Figure 1: absolute difference of dominant bacteria (families/genera) between AIS patients and control samples. Supplementary Figure 2: Venn diagrams of genus before and after treatment in the (A) control group or (B) THD group. Supplementary Figure 3: comparison of each LPS-/SCFA-producing bacterium among sample groups. Supplementary Figure 4: stroke indices of pretreatment samples in control and THD groups. (A) NIHSS scores; (B) mRS scores; (C) BI scores; (D) fire-heat scores.
