# Supplementary material for: Gut Microbiota-Related Effects of Tanhuo Decoction in Acute Ischemic Stroke
Source: Oxid Med Cell Longev. 2021 May 27;2021:5596924. doi: 10.1155/2021/5596924 (PMC8175183; doi:10.1155/2021/5596924)
Supplement: Supplementary Materials — Supplementary Table 1: batch numbers and components of the herbs in THD. Supplementary Table 2: baseline characteristics pretreatment in the control and THD groups. Supplementary Table 3: parameters of cooccurrence networks of all groups in this study. Supplementary Table 4: degrees of the genera in cooccurrence networks of patient groups in this study. Supplementary Table 5: MCC scores for Hub genera in cooccurrence networks of patient groups in this study. Supplementary Figure 1: absolute difference of dominant bacteria (families/genera) between AIS patients and control samples. Supplementary Figure 2: Venn diagrams of genus before and after treatment in the (A) control group or (B) THD group. Supplementary Figure 3: comparison of each LPS-/SCFA-producing bacterium among sample groups. Supplementary Figure 4: stroke indices of pretreatment samples in control and THD groups. (A) NIHSS scores; (B) mRS scores; (C) BI scores; (D) fire-heat scores. [file 5596924.f1.docx]

Supplementary Material

# 1 Supplementary Table

**1.1 Supplementary Table 1.** Batch numbers and components of the herbs in THD.

| Herb | Information |
| --- | --- |
| Rhei Radix et Rhizoma  (Batch number: DD1251) | **Components:**  The total content of free anthraquinone $\geq$ 0.35%, including aloe emodin (C_15_H_10_O_5_), rhein acid (C_15_H_8_O_6_), emodin (C_15_H_10_O_5_), chrysophanol (C_15_H_10_O_4_), and emodin methyl ether (C_16_H_12_O_5_).  **Extracts:**  $\geq$ 25%;  The total content of anthraquinone $\geq$1.5%, including aloe emodin (C_15_H_10_O_5_), rhein acid (C_15_H_8_O_6_), emodin (C_15_H_10_O_5_), chrysophanol (C_15_H_10_O_4_), and emodin methyl ether (C_16_H_12_O_5_).  **Examination:**  SO_2_ residue $\leq$ 150mg/kg;  Rhubarb glycoside: in the chromatogram of the test sample, the same bright blue fluorescent spots shall not be shown on the position corresponding to the reference sample of rhubarb glycoside;  Dry weight loss $\leq$ 15.0%;  Total ash content $\leq$ 10.0%. |
| Coptidis Rhizoma  (Batch number: DC6061) | **Components:**  Berberine (C_20_H_17_NO_4_) $\geq$ 5.0%;  The total content of berberine (C_20_H_17_NO_4_), coptisine (C_19_H_13_NO_4_) and palmatine (C_21_H_21_NO_4_) $\geq$ 3.3%.  **Extracts:**  $\geq$ 15.0%;  **Examination:**  SO_2_ residue $\leq$150mg/kg;  Moisture $\leq$ 12.0%;  Total ash content $\leq$ 3.5%;  Auramine O: in the chromatogram of the test sample, the spots with the color same as that of Auramine O shall not be shown on the position corresponding to the reference sample of Auramine O. |
| Forsythia  (Batch number: DD1161) | **Components:**  Forsythin (C_27_H_34_O_11_) $\geq$ 0.15%;  Forsythiasin A(C_29_H_36_O_15_) $\geq$ 0.25%.  **Examination:**  SO_2_ residue $\leq$ 150mg/kg;  Unripe forsythia suspense $\leq$ 3%;  Moisture $\leq$ 10.0%;  Total ash content $\leq$ 4.0%. |
| Bile Arisaema  (Batch number: DD3081) | **Examination:**  SO_2_ residue $\leq$ 150mg/kg;  Total ash content $\leq$ 10.0%. |
| Lophatherum Gracile  (Batch number: DC7061) | **Examination:**  SO_2_ residue $\leq$ 150mg/kg;  Moisture $\leq$ 13.0%;  Total ash content $\leq$ 11.0%. |

Note: bold, category of the information of the herb.

## 1.2 Supplementary Table 2. Baseline characteristics of pre-treatment samples in control and THD groups.

| Baseline Characteristics | Control | THD | *Q* |
| --- | --- | --- | --- |
| Male sex-no.(%) | 23(15) | 27(15) | 1 |
| Age-yr | 56.2±8.7 | 61.6±15.4 | 1 |
| Weight-kg | 72.8±13.9 | 70.5±10.8 | 1 |
| BMI-kg/m2 | 25.7±4.4 | 25.0±3.3 | 1 |
| Folic acid-ng/L | 9.9±5.4 | 7.3±2.6 | 1 |
| Vitamin B12-pmol/L | 413.7±254.0 | 384.5±296.6 | 1 |
| hcy-umol/L | 16.3±9.9 | 15.7±10.4 | 1 |
| HbA1c-% | 6.4±1.9 | 5.8±1.2 | 1 |
| Blood Glucose-mmol/L | 6.4±2.4 | 5.9±1.2 | 1 |
| Triglyceride-mmol/L | 1.7±0.9 | 1.6±0.7 | 1 |
| Cholesterol-mmol/L | 4.1±0.8 | 4.0±0.9 | 1 |
| HDLL-mmol/L | 1.1±0.2 | 1.1±0.2 | 1 |
| LDLL-mmol/L | 2.5±0.7 | 2.5±1.0 | 1 |
| CRP-mg/L | 17.5±46.4 | 8.4±8.1 | 1 |
| Creatinine-umol/L | 62.7±16.1 | 68.8±20.2 | 1 |
| Uric acid-umol/L | 302.8±66.0 | 293.7±95.4 | 1 |
| Urea-mmol/L | 4.4±1.3 | 4.8±1.6 | 1 |
| AST-IU/L | 23.9±9.8 | 23.5±6.7 | 1 |
| ALT-IU/L | 23.2±16.3 | 24.0±17.6 | 1 |
| FIB-g/L | 3.6±1.5 | 3.6±1.0 | 1 |
| DDimer-ug/mL | 1.2±2.4 | 0.7±0.9 | 1 |
| TBA-umol/L | 3.7±3.4 | 4.5±3.8 | 1 |
| AA inhibition rate-% | 85.0±20.0 | 88.6±15.2 | 1 |
| ADP inhibition rate-% | 33.6±22.6 | 46.0±32.3 | 1 |
| NIHSS score | 2.7±2.5 | 4.1±2.6 | 0.8 |
| mRS score | 2.5±1.1 | 3.2±1.0 | 0.8 |
| Fire-heat score | 17.0±6.1 | 19.0±5.0 | 1 |
| BI score | 67.9±18.7 | 52.1±22.1 | 0.7 |
| ESSEN score | 2.0±1.1 | 2.6±1.4 | 1 |
| Antiplatelet therapy | 23(23) | 27(27) | 1 |
| Lipid lowering therapy | 23(23) | 27(27) | 0.8 |

## 1.3 Supplementary Table 3. Parameters of co-occurrence networks of all groups in this study.

| Group | Complexity | Positive connection number | Negative connection number |
| --- | --- | --- | --- |
| Health | 650.226 | 55 | 20 |
| pre-control | 418.706 | 19 | 21 |
| post-control | 259.287 | 9 | 19 |
| pre-THD | 313.282 | 9 | 30 |
| post-THD | 382.380 | 13 | 32 |

Note: pre-control, pre-treatment samples in control group; post-control, post-treatment samples in control group; pre-THD, pre-treatment samples in THD group; post-THD, post-treatment samples in THD group.

## 1.4 Supplementary Table 4. Degrees of the genera in co-occurrence networks of patient groups in this study.

| Genus | Control group  Pre-treatment | | THD group | |
| --- | --- | --- | --- | --- |
|  | pre-control | post-control | pre-THD | post-THD |
| *Acetobacterium* | 0 | 0 | 0 | 1 |
| *Acidaminococcus* | 0 | 0 | 1 | 2 |
| *Actinomyces* | 1 | 0 | 0 | 1 |
| *Aeromonas* | 3 | 0 | 0 | 0 |
| *Akkermansia* | 1 | 0 | 1 | 2 |
| *Alistipes* | 2 | 2 | 3 | 5 |
| *Allobaculum* | 0 | 0 | 0 | 1 |
| *Alloscardovia* | 4 | 0 | 0 | 0 |
| *Anaerostipes* | 0 | 1 | 0 | 0 |
| *Anaerotruncus* | 2 | 0 | 0 | 0 |
| *Bacteroides* | 3 | 1 | 3 | 0 |
| *Barnesiella* | 0 | 0 | 2 | 1 |
| *Bifidobacterium* | 0 | 2 | 1 | 2 |
| *Bilophila* | 1 | 1 | 1 | 0 |
| *Blautia* | 4 | 1 | 0 | 2 |
| *Bulleidia* | 1 | 0 | 0 | 0 |
| *Butyricicoccus*   \|  \|  \|  \|  \|  \| \| --- \| --- \| --- \| --- \| --- \| \|  \|  \|  \|  \|  \| \|  \|  \|  \|  \|  \| \|  \|  \|  \|  \|  \| \|  \|  \|  \|  \|  \| \|  \|  \|  \|  \|  \| \|  \|  \|  \|  \|  \| \|  \|  \|  \|  \|  \| \|  \|  \|  \|  \|  \| \|  \|  \|  \|  \|  \| \|  \|  \|  \|  \|  \| \|  \|  \|  \|  \|  \| \|  \|  \|  \|  \|  \| \|  \|  \|  \|  \|  \| \|  \|  \|  \|  \|  \| \|  \|  \|  \|  \|  \| | 1 | 0 | 0 | 0 |
| *Butyricimonas* | 0 | 1 | 3 | 1 |
| *Catenibacterium* | 1 | 0 | 1 | 1 |
| *Christensenella* | 0 | 1 | 0 | 0 |
| *Citrobacter* | 5 | 0 | 0 | 0 |
| *Clostridium* | 6 | 1 | 7 | 3 |
| *Collinsella* | 0 | 0 | 1 | 5 |
| *Coprobacillus* | 0 | 0 | 2 | 1 |
| *Coprococcus* | 3 | 0 | 2 | 0 |
| *Dialister* | 0 | 0 | 2 | 1 |
| *Dorea* | 2 | 3 | 1 | 3 |
| *Dysgonomonas* | 0 | 0 | 2 | 2 |
| *Eggerthella* | 4 | 3 | 0 | 3 |
| *Enterococcus* | 0 | 2 | 3 | 2 |
| *Epulopiscium* | 0 | 0 | 0 | 1 |
| *Eubacterium* | 1 | 3 | 0 | 0 |
| *Faecalibacterium* | 5 | 3 | 0 | 0 |
| *Fusobacterium* | 1 | 0 | 2 | 0 |
| *Gemmiger* | 2 | 3 | 2 | 2 |
| *Granulicatella* | 3 | 0 | 0 | 0 |
| *Haemophilus* | 0 | 1 | 0 | 1 |
| *Holdemania* | 1 | 2 | 0 | 1 |
| *human* | 0 | 0 | 1 | 0 |
| *Klebsiella* | 0 | 0 | 1 | 0 |
| *Lachnobacterium* | 0 | 0 | 0 | 1 |
| *Lachnospira* | 1 | 2 | 1 | 1 |
| *Lactobacillus* | 1 | 1 | 0 | 3 |
| *Lactonifactor* | 0 | 1 | 0 | 0 |
| *Megamonas* | 2 | 0 | 0 | 1 |
| *Megasphaera* | 0 | 0 | 1 | 0 |
| *Odoribacter* | 1 | 2 | 1 | 2 |
| *Olsenella* | 0 | 0 | 0 | 1 |
| *Oscillospira* | 0 | 1 | 2 | 2 |
| *Oxalobacter* | 1 | 0 | 0 | 0 |
| *Parabacteroides* | 1 | 1 | 6 | 4 |
| *Paraprevotella* | 0 | 0 | 0 | 1 |
| *Pediococcus* | 0 | 0 | 1 | 1 |
| *Peptoniphilus* | 0 | 0 | 0 | 1 |
| *Phascolarctobacterium* | 1 | 0 | 4 | 1 |
| *Polaromonas* | 0 | 0 | 0 | 3 |
| *Prevotella* | 4 | 2 | 1 | 0 |
| *Pseudoramibacter_Eubacterium* | 1 | 3 | 0 | 1 |
| *Pyramidobacter* | 2 | 0 | 3 | 6 |
| *Roseburia* | 2 | 4 | 0 | 2 |
| *Ruminococcus* | 3 | 4 | 7 | 4 |
| *Sarcina* | 1 | 0 | 0 | 0 |
| *Shigella* | 0 | 0 | 2 | 3 |
| *SMB53* | 0 | 0 | 2 | 1 |
| *Streptococcus* | 0 | 2 | 0 | 0 |
| *Succinivibrio* | 0 | 0 | 1 | 1 |
| *Sulfurospirillum* | 0 | 0 | 0 | 1 |
| *Sutterella* | 1 | 1 | 1 | 0 |
| *Turicibacter* | 1 | 0 | 1 | 2 |
| *Veillonella* | 0 | 0 | 1 | 3 |
| *Weissella* | 0 | 1 | 1 | 0 |

Note: pre-control, pre-treatment samples in control group; post-control, post-treatment samples in control group; pre-THD, pre-treatment samples in THD group; post-THD, post-treatment samples in THD group.

## 1.5 Supplementary Table 5. MCC scores for Hub genera in co-occurrence networks of patient groups in this study

| Genus | Control group  Pre-treatment  THD group | | THD group  control group  Pre-treatment | |
| --- | --- | --- | --- | --- |
|  | pre-control | post-control | pre-THD | post-THD |
| *Acidaminococcus* | - | - | - | 2 |
| *Aeromonas* | *3* | *-* | *-* | *-* |
| *Alistipes* | - | 2 | 3 | 5 |
| *Alloscardovia* | 4 | - | - | - |
| *Anaerotruncus* | 2 | - | - | - |
| *Bacteroides* | 3 | - | 3 | - |
| *Blautia* | 4 | - | - | - |
| *Butyricimonas* | - | - | 3 | - |
| *Citrobacter* | 8 | - | - | - |
| *Clostridium* | 7 | - | 9 | 3 |
| *Collinsella* | - | - | - | 5 |
| *Coprococcus* | 3 | - | 2 | - |
| *Dialister* | - | - | 2 | - |
| *Dorea* | - | 3 | - | 3 |
| *Dysgonomonas* | - | - | 2 | - |
| *Eggerthella* | 4 | 3 | - | 3 |
| *Enterococcus* | - | 2 | 3 | - |
| *Eubacterium* | - | 3 | - | - |
| *Faecalibacterium* | 6 | 3 | - | - |
| *Gemmiger* | 2 | 3 | 2 | 2 |
| *Granulicatella* | 4 | - | - | - |
| *Holdemania* | - | 2 | - | - |
| *Lachnospira* | - | 2 | - | - |
| *Lactobacillus* | - | - | - | 3 |
| *Odoribacter* | - | 2 | - | - |
| *Oscillospira* | - | - | 2 | - |
| *Parabacteroides* | - | - | 6 | 4 |
| *Phascolarctobacterium* | - | - | 4 | - |
| Polaromona*s* | - | - | - | 3 |
| *Prevotella* | 4 | 2 | - | - |
| *Pseudoramibacter_Eubacterium* | - | 3 | - | - |
| *Pyramidobacter* | - | - | 3 | 6 |
| *Roseburia* | 2 | 4 | - | 2 |
| *Ruminococcus* | 3 | 4 | 9 | 4 |
| *Shigella* | - | - | 2 | 3 |
| *Streptococcus* | - | 2 | - | - |
| *Veillonella* | - | - | - | 3 |

Note: pre-control, pre-treatment samples in control group; post-control, post-treatment samples in control group; pre-THD, pre-treatment samples in THD group; post-THD, post-treatment samples in THD group.

# 2 Supplementary Figure

**2.1 Supplementary Figure 1.** Absolute difference of dominant bacteria (families/genera) between AIS patients and control samples. (A) Absolute difference of dominant genera between AIS patients and control samples. In THD group, the abundance of the most two abundant genera, *Prevotella* and *Bacteroides*, became closer to those of health samples, while the changes were opposite in control group. (B) Absolute difference of dominant families between AIS patients and control samples. In THD group, the abundance of the most two abundant families, *Prevotellaceae* and *Bacteroidaceae*, became closer to those of health samples, while the changes were opposite in control group. pre-control, pre-treatment samples in control group; post-control, post-treatment samples in control group; pre-THD, pre-treatment samples in THD group; post-THD, post-treatment samples in THD group. Pre-control and pre-THD were distinguished by dark grey and light grey in the figure.


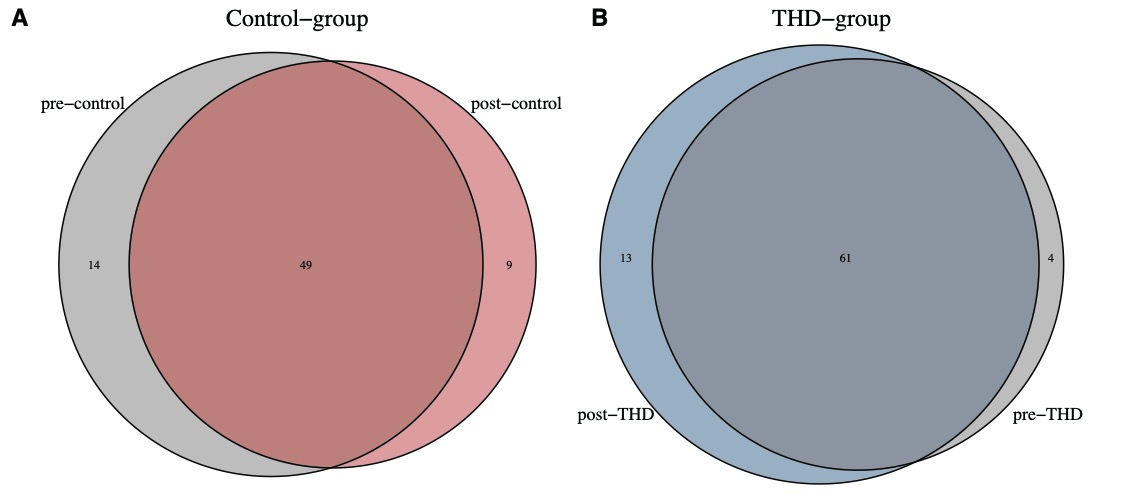


# 2.2 Supplementary Figure 2. Venn diagrams of genus before and after treatment in control group (A) and THD group (B). pre-control, pre-treatment samples in control group; post-control, post-treatment samples in control group; pre-THD, pre-treatment samples in THD group; post-THD, post-treatment samples in THD group.

**2.3 Supplementary Figure 3.** Comparison of each LPS-/SCFA-producing bacterium among sample groups. The title of each panel is named as the combination of the genus name and the SCFA name that can be produced by the genus. * *Q* ≤ 0.05, ** *Q* ≤ 0.01, *** *Q* ≤ 0.001. pre-control, pre-treatment samples in control group; post-control, post-treatment samples in control group; pre-THD, pre-treatment samples in THD group; post-THD, post-treatment samples in THD group. Pre-control and pre-THD were distinguished by dark grey and light grey in the figure.

**2.4 Supplementary Figure 4.** Indices of neurologic impairment (A), disability (B), ability (C) and syndromes of fire-heat (D) for pre-treatment samples in control and THD groups. pre-control, pre-treatment samples in control group; pre-THD, pre-treatment samples in THD group.
